# Multimorbidity and use of health services in a population diagnosed with COVID-19 in a municipality in the Southern Region of Brazil, 2020-2021: a cross-sectional study

**DOI:** 10.1590/S2237-96222024V33E2023915.EN

**Published:** 2024-02-23

**Authors:** Felipe Mendes Delpino, Yohana Pereira Vieira, Suele Manjourany Duro, Bruno Pereira Nunes, Mirelle de Oliveira Saes

**Affiliations:** 1 Universidade Federal de Pelotas, Programa de Pós-Graduação em Enfermagem, Pelotas, RS, Brazil; 2 Universidade Federal do Rio Grande, Programa de Pós-Graduação em Ciências da Saúde, Rio Grande, RS, Brazil

**Keywords:** Health Services, COVID-19, Cross-Sectional Studies, Multimorbidity, Servicios de Salud, COVID-19, Estudios Transversales, Multimorbilidad, Serviços de Saúde, Covid-19, Estudos Transversais, Multimorbidade

## Abstract

**Objective:**

To assess association between multimorbidity and use of health services in a population diagnosed with COVID-19, in southern Brazil.

**Methods:**

This was a cross-sectional study with data from a longitudinal study carried out in the city of Rio Grande, Rio Grande do Sul, Brazil, in 2021 with all adult individuals diagnosed with COVID-19; descriptive analyses were performed and presented as proportions with 95% confidence intervals (95%CI); Poisson regression was performed and reported as prevalence ratios (PR) in order to assess association between multimorbidity (3 or more diseases) and healthcare service use.

**Results:**

In total, 2,919 participants were included, of which 40.4% had multimorbidity (≥ 2 diseases); the adjusted results showed that individuals with multimorbidity were more likely to use most of the services assessed, PR = 3.21 (95%CI 1.40;7.37), for Emergency Rooms.

**Conclusion:**

Multimorbidity was associated with using different types of health services.

## Study contributions

**Main results:**

Cases with multimorbidity used services more; those with hypertension, diabetes and cardiovascular problems were associated with more consultations with cardiologists; those in pain were associated with more consultations with physiotherapists; and those with psychological disorders were associated with greater mental health service use.

**Implications for services:**

The results can help health service managers in managing and optimizing resources, allocating individuals with specific disease patterns to specialized health services, which will help to reduce waiting times and inequalities.

**Perspectives:**

Implementation of public policies aimed at management and prevention of multimorbidity could result in a reduction in the use of health services - and a reduction in costs -, in addition to improving affected people’s quality of life.

## Resumen

**Objetivo:**

Analizar la asociación entre multimorbilidad y uso de servicios de salud en una población diagnosticada con COVID-19, en el Sur de Brasil.

**Métodos:**

Estudio transversal con datos de un estudio longitudinal realizado en la ciudad de Río Grande, Rio Grande do Sul, Brasil, en el año 2021, con todos los individuos adultos diagnosticados con COVID-19; se realizaron análisis descriptivos y se presentaron como proporciones con intervalos de confianza del 95% (IC_95%_); se realizó una regresión de Poisson y se informó como razón de prevalencia (PR).

**Resultados:**

En total se incluyeron 2.919 participantes, de los cuales el 40,4% presentaba multimorbilidad (≥ 2 doenças); los resultados ajustados mostraron que los individuos con multimorbilidad tenían mayor probabilidad de utilizar la mayoría de los servicios evaluados, RP = 3,21 (IC_95%_ 1,40;7,37) para unidades Primeros auxilios.

**Conclusión:**

La multimorbilidad se asoció con el uso de diferentes tipos de servicios de salud.

## Resumo

**Objetivo:**

Analisar a associação entre multimorbidade e uso de serviços de saúde em uma população diagnosticada com covid-19, no Sul do Brasil.

**Métodos:**

Estudo transversal, utilizando-se dados de um estudo longitudinal realizado na cidade de Rio Grande, estado do Rio Grande do Sul, Brasil, em 2021, com todos os indivíduos adultos diagnosticados com covid-19; análises descritivas foram realizadas e apresentadas como proporções com intervalos de confiança de 95% (IC_95%_); a regressão de Poisson foi realizada e relatada como razão de prevalências (RP), para avaliar a associação entre multimorbidade e utilização de serviços de saúde.

**Resultados:**

Dos 2.919 participantes, 40,4% apresentavam multimorbidade (≥ 2 doenças); os resultados ajustados mostraram que indivíduos com multimorbidade (3 ou mais doenças) apresentaram maior probabilidade de utilização da maioria dos serviços avaliados (RP = 3,21; IC_95%_ 1,40;7,37) em unidades de pronto-socorro.

**Conclusão:**

A multimorbidade esteve associada à utilização de diferentes tipos de serviços de saúde.

## INTRODUCTION

Management of multiple chronic diseases, in a long-term perspective, represents a difficulty for the organization of health services, due to their complexity.^1^ ^)^ Use of health services by individuals with multiple chronic diseases derives from the need for control/ treatment, monitoring and, above all, prevention of adverse outcomes related to the clinical picture of multimorbidity.^2^

Multimorbidity is described as the presence of multiple chronic conditions, involving two or more diseases simultaneously, in the same individual.^1^ It is positively associated with age, decreased functional capacity, reduced well-being and quality of life, as well as increased mortality.^3^ A study conducted in the city of Rio de Janeiro identified that activities associated with multimorbidity were hospitalizations and appointments in primary health care services provided by the Brazilian National Health System (*Sistema Único de Saúde* - SUS).^4^

During the COVID-19 pandemic, individuals infected with SARS-CoV-2 and suffering from multimorbidity were at greater risk of developing severe forms of the disease.^5^ In Brazil, adults and the elderly showed high prevalence of multimorbidity, ranging from 18.3 to 67.8%;^6^ ^)-(^ ^9^ in particular during the pandemic, multimorbidity incidence was 27% (95%CI 23.5;31.1).^10^ Around 72% of individuals in intensive care units (ICUs) had multimorbidity,^11^ and prevalence of admissions to and deaths in ICUs grew as the number of morbidities increased.^12^ Multimorbidity, therefore, affects health service use indicators, such as hospitalizations and simultaneous use of several services at different levels of care.^13^

The most current literature shows that individuals with multimorbidity use health services more,^13^ ^),(^ ^14^ although data on this demand is scarce among publications, especially with regard to primary and secondary health care services. In the context of the COVID-19 pandemic, there is a gap in knowledge regarding the magnitude of the relationship between health service use and multimorbidity in infected individuals. Therefore, studies that investigate the relationship between multimorbidity and use of health services following SARS-CoV-2 infection, i.e. coronavirus, can be relevant in the current scenario. Furthermore, the frequency and possibility of reinfection, in addition to the emergence of what is referred to as long COVID, requires understanding of how pre-existing health conditions among those infected impact the demand for medical services, this being a crucial fact for targeting prevention, management and allocating resources more effectively and comprehensively.

The objective of this study was to assess association between multimorbidity and use of health services in a population diagnosed with COVID-19, in southern Brazil.

## METHODS

*Study design*

This was a cross-sectional study with individuals diagnosed as having COVID-19 between December 2020 and March 2021.

*Context*

We used data from the SulCovid study, conducted in the city of Rio Grande, in the extreme south of the state of Rio Grande do Sul, Brazil. Rio Grande is a port city. It covers an area of ​​2,817 km² and in 2021 had a population of 212,881 inhabitants, according to data from the Brazilian Institute of Geography and Statistics (*Instituto Brasileiro de Geografia e Estatística* - IBGE).

*Participants*

The criteria for participant inclusion in the study were: being 18 years of age or older; having been diagnosed with COVID-19 between December 2020 and March 2021, using molecular biology testing; having had COVID-19 symptoms during their illness; and having received medical care in the municipality of Rio Grande.

Cases who received treatment in the municipality but lived in other cities were excluded, as were those with functional limitations and/or advanced neurological diseases that made it impossible for them to answer the questionnaire, as well as those who were deprived of liberty. After five attempts to make contact via telephone calls and a further attempt via WhatsApp, followed by three attempts to make home visits, individuals who were not located were considered to be losses.

The Rio Grande Sanitary Surveillance service provided a list of individuals with molecular biology tests with positive COVID-19 results, including tests carried out in various locations such as pharmacies, laboratories and health services. Data collection took place from July to October 2021, 6.5 months after infection on average.

*Data sources and measurement*

We used data from the study entitled “Research to monitor the health of adults and elderly people after COVID-19 infection in Rio Grande - SulCovid-19”.

Data collection was planned in two stages: telephone collection and home collection. For telephone collection, up to five contacts were made, on alternate days and times. Individuals who did not answer any telephone calls, nor WhatsApp calls, were selected for the home visit stage. Three home visit attempts were made in order to interview those not contacted in the previous stage.

The questionnaires were administered by interviewers trained beforehand. For data collection we used electronic devices (tablets) with the REDcap platform installed. Each interview lasted approximately 15-20 minutes,^15^ ^),(^ ^16^ whereby participants had the option of answering the interviewer face-to-face.

The questionnaire, developed to be administered during telephone calls and home visits, included semi-structured questions about (i) socioeconomic variables, (ii) symptoms during and after COVID-19 infection, (iii) medical diagnosis of morbidities, (iv) behavioral characteristics and (v) use of health services.

*Variables*

The “use of health services” outcome was investigated by asking the question *“After you were infected with COVID-19, how many times did you need care in (health service)?”,* with the option to provide a continuous answer (number of times the service was used), dichotomized into “no” (no service use) and “yes” (service use one or more times).

Health services (primary healthcare center, private medical service, emergency care unit, private emergency room, emergency room, emergency services, specialist physicians, specialized services, pulmonologist, neurologist, cardiologist, psychiatrist, physiotherapist and psychologist) were analyzed by asking the question *“After you were infected with COVID-19, did you need to seek specialized care (please tick however many options you need to)”,* the reply option of which was dichotomized into: no; yes.

The outcomes were built based on a combination of “emergency service” variables (emergency care unit, private emergency room, emergency room), “specialist physician” variables (pulmonologist, neurologist, cardiologist and psychiatrist) and “specialized service” variables (physiotherapist and psychologist). All variables were equally dichotomized (no; yes), taking “yes” to mean use of at least one of the services analyzed.

The independent variable was multimorbidity, measured by counting self-reported morbidities in response to the question “*Has a doctor told you that you have...?*”, based on a list of 12 selected diseases: (i) hypertension; (ii) eye problems (cataracts, glaucoma, diabetic retinopathy and macular degeneration); (iii) arthritis or rheumatism; (iv) depression; (v) anxiety; (vi) diabetes *mellitus*; (vii) osteoporosis; (viii) heart problems; (ix) respiratory problems (emphysema, chronic bronchitis or chronic obstructive pulmonary disease, asthma); (x) cancer; (xi) urinary incontinence; and (xii) chronic illness other than these. Questions about depression and anxiety included the psychiatry and psychology specialties.

Multimorbidity was operationalized as an ordinal variable, classified into three categories: zero to one morbidity; two morbidities; three or more morbidities.^17^ ^),(^ ^18^

The following variables were used as independent covariables:

a) sex (male; female);

b) aged (in years: 18-59; 60 or over);

c) marital status (married/living with a partner; single/separated/widowed);

d) income (in BRL: BRL 0 - 1,000; BRL 1,001 - 2,000; BRL 2,001 - 4,000; BRL 4,001 or more);

e) hospitalization (no; yes);

f) body mass index (BMI: low/normal weight; overweight; obese); and

g) tobacco smoking (never smoked; smoker/former smoker).

*Statistical analyses*

Descriptive data were presented as proportions and 95% confidence intervals (95%CI). In order to identify multimorbidity patterns, principal component analysis (PCA) was performed, which allows groups of diseases to be combined based on their degree of correlation.^19^ First, an analysis was carried out without restrictions on the number of factors to be retained and then orthogonal varimax rotation was performed in order to obtain patterns that were not correlated with each other and improve data interpretation. The number of patterns to be extracted was defined based on the criterion of larger eigenvalues using T units and screeplot graphs, in which the points with the greatest slope indicate the number of factors to be considered in the analysis. Following these analyses, the model was built by setting the number of multimorbidity patterns to be retained. In order to verify the adequacy of the analysis, the Bartlett test was performed to identify whether there was correlation between the variables. The groups that contributed to the characterization of each pattern were those with factor loadings ≥ 0.3 or ≤ -0.3. The patterns were named based on the characteristics of the retained items: (i) the cardiovascular pattern - hypertension, diabetes *mellitus* and cardiovascular diseases; (ii) the musculoskeletal pattern - osteoporosis and rheumatism; and (iii) the mental disorders pattern - depression and anxiety. The p-value used in the Bartlett test was 0.000 - indicating that the variables have a significant correlation, enabling groups of diseases to be formed.

The independent variables underwent a collinearity test, using Pearson’s correlation coefficient: those that showed high collinearity with each other were discarded from the model.

In order to test the behavior of the variables in the adjustment of the regression models, subsequent analyses were performed with a hierarchical model in the following order: 1^st^ - sex, age (in years), marital status and income; 2^nd^ - smoking; 3^rd^ - BMI; and 4^th^ - hospital admission/hospitalization.

Crude and adjusted analyses comparing results and exposures were performed using Poisson regression with robust variance adjustment, reported as prevalence ratios (PR). Analyses were also performed between the patterns identified, through PCA, and the outcomes were assessed. All associations with 95%CI without overlap between categories were considered to be statistically significant. The data were analyzed using Stata 15.0 statistical software.

The study protocol was approved by the *Universidade Federal do Rio Grande* Research Ethics Committee: Opinion No. 4.375.6, dated November 3, 2020; Certificate of Submission for Ethical Appraisal (*Certificado de Apresentação para Apreciação Ética* - CAAE) No. 39081120.0.0000.5324.

## RESULTS

**Figure 1:**
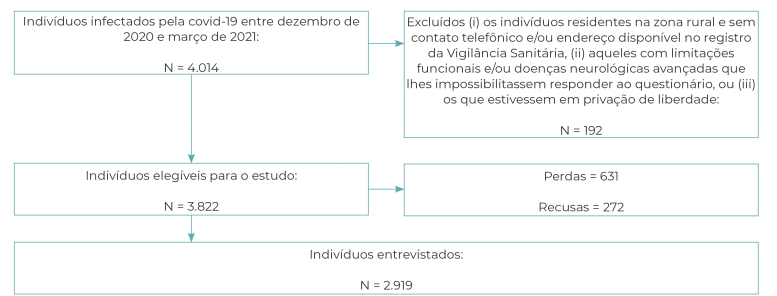
Recruitment process of the participants of the SulCovid Study, Rio Grande, Rio Grande do Sul, Brazil, 2021

Out of 4,014 individuals who tested positive for COVID-19 (Figure 1), 3,822 were eligible to participate in the study. Initially, 192 cases were excluded because they reported incomplete data, lived in a rural area and/or had no telephone contact and address available at the municipality’s Sanitary Surveillance service, had functional limitations and/or advanced neurological diseases that made it impossible for them to answer the questionnaire or because they were deprived of liberty. After losses and refusals (631 and 272, respectively), 2,919 individuals were interviewed, of which 59.6% (95%CI 57.8;61.4) did not have multimorbidity, 17.8% had two diseases (95%CI 16.5;19.3) and 22.6% had three or more chronic diseases (95%CI 21.1;24.2).

With regard to sex and age, out of the definitive sample of 2,919 participants, 59.6% were female and 83.3% were between 18 and 59 years of age (Table 1). The majority were of White/Asian race/skin color (77.9%) and reported having high school education (44.2%), 60.6% were married or lived with a partner. The study also revealed that 24.4% of participants were smokers or former smokers, and that 73.3% were overweight or obese. Regarding self-rated health, 58% of the respondents considered their health status to be good.

**Table 1:** Sociodemographic and behavioral characteristics of individuals (n = 2,919) following COVID-19 infection, Rio Grande, Rio Grande do Sul, Brazil, 2021

Characteristics	Total n (%)	Multimorbidade n (%)
Sex		
Male	1,208 (41.4)	271 (26.1)
Female	1,711 (59.6)	768 (73.9)
Age (in years)		
18-59	2,420 (83.3)	726 (70.2)
≥ 60	482 (16.7)	308 (29.8)
Schooling		
No schooling	15 (0.5)	353 (35.0)
Elementary education	713 (24.9	376 (37.3)
High school education	1,264 (44.2)	279 (27.7)
Higher education	871 (30.4)	
Marital status		
Married/living with a partner	1,757 (60.6)	613 (59.5)
Single/separated/divorced	1,144 (39.4)	418 (40.5)
***Per capita* income (in BRL)**		
0 - 1,000	668 (26.1)	283 (30.3)
1,001 - 2,000	995 (38.9)	374 (40.0)
2,001 - 4,000	604 (23.6)	190 (20.3)
4,001 or more	288 (11.4)	88 (9.4)
Smoking		
Never smoked	2,197 (75.6)	723 (69.6)
Smoker/former smoker	708 (24.4)	316 (30.4)
Body Mass Index (BMI)		
Low/normal weight	757 (26.7)	223 (22.2)
Overweight/obese	2,076 (73.3)	781 (77.8)
Hospitalization		
No	2,307 (96.3)	722 (92.9)
Yes	88 (3.7)	55 (7.1)

Compared to those who did not have multimorbidities, those who had two diseases showed greater use of the following health services: private medical service (PR = 1.41; 95%CI 1.15;1.73); emergency care unit (PR = 1.57; 95%CI 1.10;2.25); emergency room (PR = 3.24; 95%CI 1.45;7.22); emergency services (PR = 1.62; 95%CI 1.21;2.18); specialist physicians (PR = 2.34; 95%CI 2.04;4.13); specialized services (PR = 2.91; 95%CI 1.88;2.95); neurologist (PR = 2.51; 95%CI 1.08;5.83); cardiologist (PR = 2.04; 95%CI 1.41;2.95); psychiatrist (PR = 5.02; 95%CI 2.74;9.20); physiotherapist (PR = 2.26; 95%CI 1.08;4.71); and psychologist (PR = 3.30; 95%CI 2.08;5.23) (Table 2). In the case of multimorbidity involving three or more diseases, risk of using all the health services was even greater. Services that were not associated with the presence of two diseases became associated when there was the presence of three or more diseases, such as primary healthcare centers (PR = 1.47; 95%CI 1.23;1.77), private emergency rooms (PR = 3.09; 95%CI 1.78;5.36) and consultations with a pulmonologist (PR = 1.81; 95%CI 1.12;2.91).

**Table 2:** Adjusted analysis of association between multimorbidity and use of health services in individuals (n = 2,919) following COVID-19 infection, Rio Grande, Rio Grande do Sul, Brazil, 2021

Health services	Crude PR^a^ (95%CI^b^)	Crude PR^a^ (95%CI^b^)	p-value	Adjusted PR^a^ (95%CI^b^)	Adjusted PR^a^ (95%CI^b^)	p-value
	2 diseases	≥ 3 diseases		2 diseases	≥ 3 diseases	
Primary healthcare center	1.25 (1.06;1.47)	1.66 (1.45;1.90)	< 0.001	1.15 (0.93;1.42)	1.47 (1.23;1.77)	< 0.001
Private medical service	1.45 (1.24;1.70)	1.52 (1.32;1.76)	< 0.001	1.41 (1.15;1.73)	1.69 (1.40;2.04)	< 0.001
Emergency care unit	1.37 (1.02;1.85)	1.82 (1.40;3.36)	< 0.001	1.57 (1.10;2.25)	1.51 (1.05;2.17)	< 0.001
Private emergency room	1.55 (0.94;2.55)	2.18 (1.42;3.35)	< 0.001	1.49 (0.76;2.93)	3.09 (1.78;5.36)	< 0.001
Emergency room	2.27 (1.25;4.13)	3.60 (2.16;6.00)	< 0.001	3.24 (1.45;7.22)	3.21 (1.40;7.37)	< 0.001
Emergency services^c^	1.50 (1.18;1.90)	2.09 (1.70;2.56)	< 0.001	1.62 (1.21;2.18)	1.82 (1.36;2.42)	< 0.001
Specialist physicians^d^	2.41 (1.99;2.92)	3.37 (2.85;3.98)	< 0.001	2.34 (2.04;4.13)	2.14 (1.44;3.20)	< 0.001
Specialized services^e^	3.22 (2.45;4.24)	2.60 (1.95;3.46)	< 0.001	2.91 (1.88;2.95)	3.04 (2.47;3.76)	< 0.001
Pulmonologist	2.05 (1.47;2.87)	2.41 (1.77;3.28)	< 0.001	1.67 (0.99;2.81)	1.81 (1.12;2.91)	< 0.001
Neurologist	2.14 (1.22;3.74)	3.74 (2.35;5.93)	< 0.001	2.51 (1.08;5.83)	2.97 (1.35;6.51)	< 0.001
Cardiologist	2.25 (1.73;2.92)	4.15 (3.36;5.12)	< 0.001	2.04 (1.41;2.95)	3.82 (2.78;5.24)	< 0.001
Psychiatrist	5.20 (3.22;8.41)	4.92 (3.05;7.92)	< 0.001	5.02 (2.74;9.20)	6.09 (3.21;11.5)	< 0.001
Physiotherapist	1.93 (1.16;3.21)	2.29 (1.43;3.65)	< 0.001	2.26 (1.08;4.71)	2.40 (1.20;4.84)	< 0.001
Psychologist	4.34 (3.07;6.14)	3.42 (2.39;4.91)	< 0.001	3.30 (2.08;5.23)	3.89 (2.45;6.17)	< 0.001

a) PR: Prevalence ratio; b) 95%CI: 95% Confidence interval; c) Emergency services: emergency care unit, private emergency room and emergency room; d) Specialist physicians: pulmonologist, neurologist, cardiologist and psychiatrist; e) Specialized services: physiotherapist and psychologist.

Note: Adjusted for sex, age (in years), marital status, income, hospitalization, BMI and smoking.

The principle components analysis resulted in the definition of three disease patterns:

a) Pattern 1 [31.1% (95%CI 29.7;33.1)], comprised of hypertension, diabetes *mellitus* and cardiovascular diseases;

b) Pattern 2 [13.3% (95%CI 12.1;14.6)], comprised of osteoporosis and rheumatism;

c) Pattern 3 [33.5% (95%CI 31.8;35.2)], comprised of depression and anxiety.

Although most health services were associated with the three disease patterns (Table 3), it was possible to note that Pattern 1 (hypertension, diabetes *mellitus* and cardiovascular diseases) had a greater association with consultations with a cardiologist (PR = 5.02; 95% CI 3.75;6.70), compared to Patterns 2 and 3. Pattern 2 (osteoporosis and rheumatism) was the only one to be associated with consultations with a physiotherapist (PR = 2.26; 95%CI 1.20;4.27), this being a medical specialty with which Patterns 1 and 3 showed no association. However, the strong association of Pattern 3 (depression and anxiety) with psychiatric consultations (PR = 8.80; 95%CI 4.86;15.9) and psychological consultations (PR = 5.53; 95%CI 3.63;8.41) is noteworthy.

**Table 3:** Adjusted analysis of association between patterns of chronic diseases and use of health services in individuals (n = 2,919) following COVID-19 infection, Rio Grande, Rio Grande do Sul, Brazil, 2021

Health services	Pattern 1				Pattern 2				Pattern 3			
	Crude PR^a^ (95%CI^b^)	p-value	Adjusted PR^a^ (95%CI^b^)	p-value	Crude PR^a^ (95%CI^b^)	p-value	Adjusted PR^a^ (95%CI^b^)	p-value	Crude PR^a^ (95%CI^b^)	p-value	Adjusted PR^a^ (95%CI^b^)	p-value
Primary healthcare center	1,43 (1,27; 1,61)	<0,001	1,36 (1,18; 1,58)	<0,001	1,48 (1,28; 1,71)	<0,001	1,44 (1,20; 1,73)	<0,001	1,29 (1,15; 1,46)	<0,001	1,13 (0,97; 1,32	0.140
Private medical service	1,23 (1,08; 1,40)	<0,001	1.29 (1,08; 1,48)	0,010	1,43 (1,23; 1,66)	<0,001	1,24 (1,02; 1,53)	0,070	1,37 (1,21; 1,55)	<0,001	1,45 (1,24; 1,70)	<0.001
Emergency care unit	1,49 (1,19; 1,87)	<0,001	1,17 (0.87; 1.56)	0,650	1,19 (0,88; 1,62)	0,260	-	0,490	1,61 (1,29; 2,00)	<0,001	1,37 (1,04; 1,81)	0.030
Private emergency room	1,60 (1,10; 2,35)	0,020	2,02 (1,26; 3,23)	<0,001	2,27 (1,48; 3,47)	<0,001	2,74 (1,63; 4,62)	<0,001	1,52 (1,04; 2,21)	0,030	1,82 (1,13; 2,93)	0.040
Emergency room	1,55 (0,99; 2,43)	0,050	1,22 (0,64; 2,30)	0,620	1,97 (1,18; 3,31)	0,010	1,30 (0,61; 2,82)	0,480	2,14 (1,38; 3,33)	<0,001	1,69 (0,92; 3,13)	0.070
Emergency services^c^	1,56 (1,30; 1,87)	<0,001	1,37 (1,09; 1,73)	0,020	1,56 (1,25; 1,95)	<0,001	1,22 (0,90; 1,64)	0,280	1,64 (1,38; 1,96)	<0,001	1,42 (1,14; 1,79)	<0.001
Specialist physicians^d^	2,77 (2,40; 3,20)	<0,001	2,76 (2,27; 3,36)	<0,001	1,99 (1,69; 2,34)	<0,001	1,80 (1,44; 2,24)	<0,001	1,81 (1,57; 2,09)	<0,001	1,89 (1,55; 2,30)	<0.001
Specialized services^e^	1,32 (1,05; 1,67)	0,020	1,08 (0,78; 1,48)	0,640	1,77 (1,34; 2,32)	<0,001	1,41 (0,97; 2,05)	0,090	3,20 (2,53; 4,04)	<0,001	2,95 (2,18; 4,00)	<0.001
Pulmonologist	1,63 (1,25; 2,12)	<0,001	1,09 (0,72; 1,63)	0,860	1,85 (1,36; 2,52)	<0,001	1,67 (1,6; 2,62)	0,150	1,16 (0,89; 1,53)	0,270	-	-
Neurologist	3,09 (2,06; 4,66)	<0,001	1,64 (0,89; 3,05)	0,250	2,65 (1,72; 4,09)	<0,001	1,34 (0,68; 2,66)	0,650	2,40 (1,61; 3,58)	<0,001	2,25 (1,25; 4,06)	<0.001
Cardiologist	4,98 (4,07; 6,09)	<0,001	5,38 (4,10; 7,05)	<0,001	2,40 (1,97; 2,92)	<0,001	2,07 (1,57; 2,73)	<0,001	1,53 (1,27; 1,84)	<0,001	1,55 (1,21; 1,99)	<0.001
Psychiatrist	1,18 (0,81; 1,73)	0,390	1,07 (0,65; 1,75)	0,840	1,46 (0,90; 2,30)	0,130	1,33 (0,76; 2,25)	0,650	11,17 (6,70; 18,59)	<0,001	9,27 (5,23; 16,40)	<0.001
Physiotherapist	2,37 (1,60; 3,52)	<0,001	1,85 (1,05; 3,26)	0,150	3,33 (2,22; 5,00)	<0,001	2,50 (1,41; 4,42)	0,010	1,48 (1,00; 2,20)	0,050	-	-
Psychologist	1,17 (0,88; 1,58)	0,280	0,94 (0,63; 1,39)	0,930	1,15 (0,77; 1,70)	0,500	0,77 (0,43; 1,36)	0,720	6,16 (4,43; 8,58)	<0,001	5,80 (3,86; 8,70)	<0.001

a) PR: Prevalence ratio; b) 95%CI: 95% Confidence interval; c) Emergency services: emergency care unit, private emergency room and emergency room; d) Specialist physicians: pulmonologist, neurologist, cardiologist and psychiatrist; e) Specialized services: physiotherapist and psychologist.

Notes: Pattern 1: hypertension, diabetes *mellitus* and cardiovascular diseases; Pattern 2: osteoporosis and rheumatism; Pattern 3: depression and anxiety.

Adjusted for sex, age (in years), marital status, income, hospitalization, body mass index (BMI) and smoking.

Table 4 presents the adjusted analysis of association between multimorbidity, in a dichotomous manner, and use of health services. With the exception of use of emergency rooms, all the analyses were statistically significant, showing that participants with multimorbidity were more likely to use the services we assessed, especially cardiologists (PR = 4.63; 95%CI 3.07;6.98) and psychiatrists (PR = 4.92; 95%CI 2.42;9.99).

**Table 4:** Adjusted analysis of association between multimorbidity (dichotomized) and use of health services in individuals (n = 2,919) following COVID-19 infection, Rio Grande, Rio Grande do Sul, Brazil, 2021

Health services	Multimorbidity Adjusted PR^a^ (95%CI^b^)
Primary healthcare center	1.34 (1.14;1.58)
Private medical service	1.57 (1.32;1.62)
Emergency care unit	1.63 (1.18;1.73)
Private emergency room	2.12 (1.22;3.72)
Emergency room	2.00 (0.95;4.25)
Emergency services^c^	1.63 (1.26;2.12)
Specialist physicians^d^	3.60 (2.67;4.85)
Specialized services^e^	2.66 (1.81;3.91)
Pulmonologist	1.70 (1.09;2.66)
Neurologist	3.00 (1.28;7.08)
Cardiologist	4.63 (3.07;6.98)
Psychiatrist	4.92 (2.42;9.99)
Physiotherapist	2.85 (1.37;5.91)
Psychologist	2.95 (1.82;4.78)

a) PR: Prevalence ratio; b) 95%CI: 95% Confidence interval; c) Emergency services: emergency care unit, private emergency room and emergency room; d) Specialist physicians: pulmonologist, neurologist, cardiologist and psychiatrist; d) Specialized services: physiotherapist and psychologist.

Note: Adjusted for sex, age (in years), marital status, income, hospitalization, body mass index (BMI) and smoking.

The results of the supplementary analysis (Supplementary Table 1) showed that, regardless of overweight or obesity, multimorbidity was associated with greater use of health services.

In general terms, the results in Supplementary Tables 2 and 3 were similar to the original models, presented in Tables 2 and 3.

It was also possible to note that, regarding the use of some of the services - specialist physicians, specialized services, psychiatrists, psychologists -, Pattern 3 was associated with multimorbidity only among participants in the 18-59 and 60-69 age group, but not among those aged 70 or over (Supplementary Table 4).

## DISCUSSION

In this study, different forms of measurement made it possible to analyze association of multimorbidity with the use of different types of health services by people with COVID-19. Having several chronic diseases was associated with greater use of services. By means of principal components analysis, three patterns of multimorbidity associated with different types of health services were identified. In particular, Pattern 3, comprised of mental diseases, presented the highest prevalence ratio for psychiatric consultations and psychological consultations. Pattern 1 also stood out, presenting a high prevalence ratio for consultations with a cardiologist.

The results can offer important contributions to public health in Brazil. By understanding how pre-existing health conditions influence the need for post-infection medical care, the SUS can be better prepared to deal with future demands. The conditions present in an individual can be interconnected, in a way that requires coordinated and comprehensive health care and management. Identification of disease patterns and their association with use of health services makes it possible to target care according to each person’s health conditions.

This study has limitations, including measurement of multimorbidity using self-reporting, which may lead to a result that is not as accurate as measurement using objective methods, such as electronic medical records or medical examinations. This problem may be further aggravated by the fact that the participants had COVID-19, which may be associated (in some cases) with memory loss. This hypothesis is confirmed by a meta-analysis which found memory problems in around 27% of participants in the 19 studies it analyzed.^20^ As this is a cross-sectional study, the possibility of reverse causality also needs to be addressed. However, chronic diseases are conditions that occur throughout life, while the use of health services was assessed following diagnosis of COVID-19, with the possibility of reducing the risk of reverse causality, without however eliminating the risk. Furthermore, use of health services may be underestimated: it was assessed during the pandemic, when the majority of services were diverted to caring for COVID-19 cases.

COVID-19 may have worsened these numbers even further. Studies have shown high prevalence of mental disorders, such as anxiety,^21^ and restricting access to health services, this being inevitable given the emergence of the pandemic, could worsen this condition. Therefore, it is possible that in the coming years, health service use involving consultations with psychiatrists and psychologists will increase considerably, placing a burden on healthcare systems. Furthermore, uncertainty about the consequences of the pandemic and the lack of commitment on the part of some government officials in the country could possibly trigger the occurrence of new cases of depression and anxiety, resulting in greater use of specialized health services.

Association between multimorbidity and use of healthcare services has been demonstrated.^13^ Healthcare costs are also higher in individuals with multimorbidity, and can be up to 5.5 times greater than healthcare costs for individuals without multimorbidity; furthermore, each additional illness can increase the number of consultations by 3.2 times, and costs by 33%.^22^

The results of this study serve as a warning for primary healthcare interventions aimed at reducing future expenditure on multimorbidity management. The main finding of this study is that individuals with multimorbidity are those who most use the health services assessed. A possible explanation for this scenario lies in the fact that multimorbidity, regardless of the country’s income and gender, is associated with hospitalization and recurring hospital admission of the elderly.^23^ The complexity of multimorbidity is associated with both use of primary healthcare services and care provided in emergency rooms, which can culminate in cases of hospitalization and readmission to hospital.

The results of this research point to individuals with multimorbidity as being those who most use the majority of health services we assessed,^24^ instead of seeking care in primary healthcare services. Health System users see emergency rooms as a possibility of being quickly attended to and undergoing examinations immediately, due to the availability of resources and teams trained to deal with medical emergencies. However, in many cases, care provision in primary healthcare services would be more appropriate for the management of chronic conditions, aiming to achieve a more comprehensive and continuous approach. It should be added that individuals with multimorbidity may require greater levels of care, with regard to the most serious cases resulting from COVID-19,^25^ implying greater use of emergency rooms.

The clusters found in this study can be fundamental for guiding health service managers and making health decisions. The complexity of the interaction between one disease and another is reflected in the adverse effects it can cause. A study conducted in London, with some 826,000 medical records covering the period from 2005 to 2020, identified mental, cardiovascular, pain and liver health clusters associated with an increase in primary care consultations.^26^ The authors of that study also identified that on average individuals with multimorbidity had 12 primary care consultations.^26^ In order to prevent interactions between different morbidities, identification of clusters of diseases in primary healthcare services can can enable referral of Health System users to specialized services, avoiding unnecessary consultations with other public health professionals.

Multimorbidity is a complex and interrelated condition, requiring a multi-level approach, focusing on specific issues, such as underlying biological mechanisms and determining socioeconomic factors, for example.^2^ Multimorbidity care must focus on multidisciplinary care. Identifying clusters of diseases can be an additional tool for health professionals, contributing to the management of care by specialist physicians in a given cluster. It is necessary to create health policies capable of dealing with individuals diagnosed with multimorbidity, respecting their characteristics and prioritizing their quality of life.

Although multimorbidity prevalence is higher in high-income countries,^27^ the main problem may lie in low- and middle-income countries, a reality in which there is possibly less access to medical diagnosis, greater socioeconomic inequalities and poorer quality of care for people with multimorbidity. It is estimated that around 46% of cases of diabetes *mellitus* (approximately 175 million people) are underdiagnosed, and among these, 83.3% live in low- and middle-income countries.^28^ These are, therefore, two major challenges for health service managers, especially in low- and middle-income countries: the first challenge being expanding access to health services, while the second challenge relates to identifying clusters and referring them to specialized health professionals or multidisciplinary health teams, as appropriate.

In conclusion, multimorbidity was associated with use of different types of health services. Patterns consisting of hypertension, diabetes *mellitus* and cardiovascular problems were more associated with consultations with cardiologists; pain-related patterns were associated with the use of physiotherapy services; while the psychological disorder pattern was associated with mental health services. The results of this study, based on the disease patterns observed, provide support for health service managers and health professionals in the management of multimorbidity and in the redirection of health care, contributing to the adaptation of resources and specialties to the identified prevalence of demand, in addition to promoting of comprehensiveness between the different services offered by the SUS. Although the study was carried out during the COVID-19 pandemic, its results can contribute to prevention and management, improved efficiency and access to care for chronic conditions.

## Supplementary Table 1

### - Adjusted analysis of association between dichotomized multimorbidirty and use of health services stratified by body mass index in individuals (n = 2,919) following COVID-19 infection, Rio Grande, Rio Grande do Sul, Brazil, 2021

**Supplementary Table 1:** - Adjusted analysis of association between dichotomized multimorbidirty and use of health services stratified by body mass index in individuals (n = 2,919) following COVID-19 infection, Rio Grande, Rio Grande do Sul, Brazil, 2021

Health services	Low - normal weight (95%CI^a^)	Overweight (95%CI^a^)	Obesity (95%CI^a^)
Primary healthcare center	1.21 (0.90;1.62)	0.73 (0.53;1.01)	1.57 (1.17;2.11)
Private medical service	1.53 (1.10;2.12)	1.35 (1.04;1.74)	1.96 (1.34;2.87)
Emergency care unit	2.14 (1.14;4.01)	1.58 (0.94;2.64)	0.48 (0.24;0.95)
Private emergency room	0.25 (0.08;0.85)	2.63 (1.08;6.48)	4.21 (0.98;18.06)
Emergency room	-	-	2.00 (0.71;5.60)
Emergency services^b^	1.71 (1.07;2.73)	1.63 (1.07;2.50)	1.51 (0.94;2.41)
Specialist physicians^c^	2.68 (1.52;4.72)	3.48 (2.22;5.43)	4.93 (2.73;8.92)
Specialized services^d^	3.88 (1.76;8.56)	2.20 (1.27;3.79)	2.99 (1.46;6.14)
Pulmonologist	10.26 (3.24;32.49)	1.61 (0.85;3.05)	2.87 (1.03;7.99)
Neurologist	-	2.41 (0.80;7.25)	2.97 (0.70;12.59)
Cardiologist	4.11 (1.72;9.80)	4.21 (2.32;7.64)	5.72 (2.72;12.01)
Psychiatrist	12.66 (1.60;99.88)	3.54 (1.32;9.51)	4.14 (1.27;13.55)
Physiotherapist	2.89 (1.00;8.35)	3.82 (1.16;12.56)	3.19 (0.73;13.90)
Psychologist	8.83 (2.09;37.23)	2.00 (1.04;2.85)	2.89 (1.22;6.81)

a) 95%CI: 95% Confidence interval; b) Emergency services: emergency care unit, private emergency room and emergency room; c) Specialist physicians: pulmonologist, neurologist, cardiologist and psychiatrist; d) Specialized services: physiotherapist and psychologist.

Note: Adjusted for sex, age (in years), marital status, income, hospitalization, body mass index (BMI) and smoking.

Refers to occasions when the sample size was small and it was not possible to perform the analyses.

**Supplementary Table 2:** - Adjusted analysis of association between multimorbidity and use of health services in individuals (n = 2,919) following COVID-19 infection, Rio Grande, Rio Grande do Sul, Brazil, 2021

Health services	Crude PR^a^ (95%CI^b^)		p-value	Adjusted PR^a^ (95%CI^b^)		p-value
	2 diseases	≥ 3 diseases		2 diseases	≥ 3 diseases	
Primary healthcare center	1.25 (1.06;1.47)	1.66 (1.45;1.90)	< 0.001	1.17 (0.96;1.43)	1.48 (1.25;1.75)	< 0.001
Private medical service	1.45 (1.24;1.70)	1.52 (1.32;1.76)	< 0.001	1.39 (1.14;1.70)	1.56 (1.29;1.88)	< 0.001
Emergency care unit	1.37 (1.02;1.85)	1.82 (1.40;3.36)	< 0.001	1.48 (1.04;2.09)	1.40 (0.99;1.98)	< 0.001
Private emergency room	1.55 (0.94;2.55)	2.18 (1.42;3.35)	< 0.001	1.80 (0.95;3.41)	3.17 (1.89;5.32)	< 0.001
Emergency room	2.27 (1.25;4.13)	3.60 (2.16;6.00)	< 0.001	2.32 (1.11;4.85)	2.30 (1.09;4.86)	< 0.001
Emergency services^c^	1.50 (1.18;1.90)	2.09 (1.70;2.56)	< 0.001	1.58 (1.18;2.10)	1.75 (1.33;2.29)	< 0.001
Specialist physicians^d^	2.41 (1.99;2.92)	3.37 (2.85;3.98)	< 0.001	2.41 (1.87;3.10)	3.32 (2.63;4.19)	< 0.001
Specialized services^e^	3.22 (2.45;4.24)	2.60 (1.95;3.46)	< 0.001	2.74 (1.94;3.86)	2.00 (1.37;2.93)	< 0.001
Pulmonologist	2.05 (1.47;2.87)	2.41 (1.77;3.28)	< 0.001	1.47 (0.88;2.47)	1.77 (1.13;2.77)	< 0.001
Neurologist	2.14 (1.22;3.74)	3.74 (2.35;5.93)	< 0.001	1.55 (0.64;3.73)	1.72 (0.79;3.75)	< 0.001
Cardiologist	2.25 (1.73;2.92)	4.15 (3.36;5.12)	< 0.001	2.12 (1.50;2.98)	3.87 (2.88;5.20)	< 0.001
Psychiatrist	5.20 (3.22;8.41)	4.92 (3.05;7.92)	< 0.001	5.15 (2.97;8.90)	4.43 (2.48;7.90)	< 0.001
Physiotherapist	1.93 (1.16;3.21)	2.29 (1.43;3.65)	< 0.001	1.73 (0.85;3.52)	1.69 (0.84;3.42)	< 0.001
Psychologist	4.34 (3.07;6.14)	3.42 (2.39;4.91)	< 0.001	3.58 (2.37;5.41)	2.67 (2.36;5.24)	< 0.001

a) PR: Prevalence ratio; b) 95%CI: 95% Confidence interval; c) Emergency services: emergency care unit, private emergency room and emergency room; d) Specialist physicians: pulmonologist, neurologist, cardiologist and psychiatrist; e) Specialized services: physiotherapist and psychologist.

Note: Adjusted for sex, age (in years), marital status, income, hospitalization, body mass index (BMI) and smoking.

**Supplementary Table 3:** - Adjusted analysis of association between patterns of chronic diseases and use of health services in individuals (n = 2,919) following COVID-19 infection, Rio Grande, Rio Grande do Sul, Brazil, 2021

Health services	Pattern 1				Pattern 2				Pattern 3			
	Crude PR^a^ (95%CI^b^)	p-value	Adjusted PR^a^ (95%CI^b^)	p-value	Crude PR^a^ (95%CI^b^)	p-value	Adjusted PR^a^ (95%CI^b^)	p-value	Crude PR^a^ (95%CI^b^)	p-value	Adjusted PR^a^ (95%CI^b^)	p-value
Primary healthcare center	1.43 (1.27;1.61)	< 0.001	1.36 (1.18;1.58)	< 0.001	1.48 (1.28;1.71)	< 0.001	1.44 (1.20;1.73)	< 0.001	1.29 (1.15;1.46)	< 0.001	1.13 (0.97;1.32)	0.140
Private medical service	1.23 (1.08;1.40)	< 0.001	1.29 (1.08;1.48)	0.010	1.43 (1.23;1.66)	< 0.001	1.24 (1.02;1.53)	0.07	1.37 (1.21;1.55)	< 0.001	1.45 (1.24;1.70)	<0.001
Emergency care unit	1.49 (1.19;1.87)	< 0.001	1.17 (0.87;1.56)	0.650	1.19 (0.88;1.62)	0.260	-	0.49	1.61 (1.29;2.00)	< 0.001	1.37 (1.04;1.81)	0.030
Private emergency room	1.60 (1.10;2.35)	0.020	2.02 (1.26;3.23)	< 0.001	2.27 (1.48;3.47)	< 0.001	2.74 (1.63;4.62)	< 0.001	1.52 (1.04;2.21)	0.030	1.82 (1.13;2.93)	0.040
Emergency room	1.55 (0.99;2.43)	0.050	1.22 (0.64;2.30)	0.620	1.97 (1.18;3.31)	0.010	1.30 (0.61;2.82)	0.480	2.14 (1.38;3.33)	< 0.001	1.69 (0.92;3.13)	0.070
Emergency services^c^	1.56 (1.30;1.87)	< 0.001	1.37 (1.09;1.73)	0.020	1.56 (1.25;1.95)	< 0.001	1.22 (0.90;1.64)	0.280	1.64 (1.38;1.96)	< 0.001	1.42 (1.14;1.79)	< 0.001
Specialist physicians^d^	2.77 (2.40;3.20)	< 0.001	2.76 (2.27;3.36)	< 0.001	1.99 (1.69;2.34)	< 0.001	1.80 (1.44;2.24)	< 0.001	1.81 (1.57;2.09)	< 0.001	1.89 (1.55;2.30)	< 0.001
Specialized services^e^	1.32 (1.05;1.67)	0.020	1.08 (0.78;1.48)	0.640	1.77 (1.34;2.32)	< 0.001	1.41 (0.97;2.05)	0.090	3.20 (2.53;4.04)	< 0.001	2.95 (2.18;4.00)	< 0.001
Pulmonologist	1.63 (1.25;2.12)	< 0.001	1.09 (0.72;1.63)	0.860	1.85 (1.36;2.52)	< 0.001	1.67 (1.6;2.62)	0.150	1.16 (0.89;1.53)	0.270	-	-
Neurologist	3.09 (2.06;4.66)	< 0.001	1.64 (0.89;3.05)	0.250	2.65 (1.72;4.09)	< 0.001	1.34 (0.68;2.66)	0.650	2.40 (1.61;3.58)	< 0.001	2.25 (1.25;4.06)	< 0.001
Cardiologist	4.98 (4.07;6.09)	< 0.001	5.38 (4.10;7.05)	< 0.001	2.40 (1.97;2.92)	< 0.001	2.07 (1.57;2.73)	< 0.001	1.53 (1.27;1.84)	< 0.001	1.55 (1.21;1.99)	< 0.001
Psychiatrist	1.18 (0.81;1.73)	0.390	1.07 (0.65;1.75)	0.840	1.46 (0.90;2.30)	0.130	1.33 (0.76;2.25)	0.650	11.17 (6.70;18.59)	< 0.001	9.27 (5.23;16.40)	< 0.001
Physiotherapist	2.37 (1.60;3.52)	< 0.001	1.85 (1.05;3.26)	0.150	3.33 (2.22;5.00)	< 0.001	2.50 (1.41;4.42)	0.010	1.48 (1.00;2.20)	0.050	X	X
Psychologist	1.17 (0.88;1.58)	0.280	0.94 (0.63;1.39)	0.930	1.15 (0.77;1.70)	0.500	0.77 (0.43;1.36)	0.720	6.16 (4.43;8.58)	< 0.001	5.80 (3.86;8.70)	< 0.001

a) PR: Prevalence ratio; b) 95%CI: 95% Confidence interval; c) Emergency services: emergency care unit, private emergency room and emergency room; d) Specialist physicians: pulmonologist, neurologist, cardiologist and psychiatrist; e) Specialized services: physiotherapist and psychologist.

Notes: Pattern 1: hypertension, diabetes *mellitus* and cardiovascular diseases; Pattern 2: osteoporosis and rheumatism; Pattern 3: depression and anxiety.

Adjusted for sex, age (in years), marital status, income, hospitalization, body mass index (BMI) and smoking.

Refers to occasions when the sample size was small and it was not possible to perform the analyses.

**Supplementary Table:** 4 - Adjusted analysis of association between patterns of multimorbidity and use of health services stratified by age group in individuals (n = 2,919) following COVID-19 infection, Rio Grande, Rio Grande do Sul, Brazil, 2021

Health services	Pattern 1			Pattern 2			Pattern 3		
	18-59 (95%CI^a^)	60-69 (95%CI^a^)	≥ 70 (95%CI^a^)	18-59 (95%CI^a^)	60-69 (95%CI^a^)	≥ 70 (95%CI^a^)	18-59 (95%CI^a^)	60-69 (95%CI^a^)	≥ 70 (95%CI^a^)
Primary healthcare center	1.32 (1.11;1.57)	1.36 (0.87;2.11)	2.05 (0.70;5.99)	1.44 (1.14;1.82)	0.90 (0.57;1.43)	1.85 (1.04;3.29)	1.14 (0.99;1.31)	1.41 (0.94;2.12)	1.57 (1.01;2.45)
Private medical service	1.18 (0.97;1.43)	1.84 (1.08;3.15)	0.51 (0.31;0.83)	1.32 (1.03;1.70)	1.24 (0.74;2.07)	0.98 (0.57;1.68)	1.38 (1.16;1.65)	2.05 (1.32;3.17)	1.81 (1.12;2.94)
Emergency care unit	1.29 (1;07;1.54)	2.35 (0.77;7.10)	2.77 (1.13;6.82)	1.3 (0.94;1.88)	0.32 (0.09;1.13)	0.81 (0.28;2.35)	1.28 (0.94;1.74)	1.84 (0.86;3.93)	2.79 (0.98;7.91)
Private emergency room	2.19 (1.29;3.72)	3.88 (1.88;7.94)	-	2.53 (1.31;4.89)	2.82 (0.66;12.10)	-	1.58 (0.95;2.64)	-	-
Emergency room	5.65 (1.93;16.4)	3.61 (0.74;17.6)	-	2.22 (1.00;4.91)	0.47 (0.06;3.36)	-	1.84 (0.93;3.66)	-	-
Emergency services^b^	1.46 (1.12;1.90)	0.58 (0.30;1.10)	-	1.36 (0.95;1.95)	0.70 (0.31;1.61)	1.13 (0.46;2.76)	1.34 (1.05;1.72)	1.85 (1.00;3.43)	2.49 (1.04;5.98)
Specialist physicians^c^	2.68 (2.14;3.35)	3.03 (1.54;5.99)	2.53 (1.33;4.79)	1.90 (1.42;2.52)	1.35 (0.81;2.26)	1.23 (0.78;1.92)	2.05 (1.62;2.60)	1.63 (1.01;2.65)	1.29 (0.87;1.90)
Specialized services^d^	1.75 (1.14;2.68)	1.68 (1.07;2.62)	0.15 (0.03;0.63)	1.68 (1.07;2.62)	0.50 (0.13;1.85)	2.12 (0.75;6.02)	3.11 (2.23;4.34)	4.45 (1.58;12.51)	1.72 (0.72;4.15)
Pulmonologist	10.2 (5.56;18.9)	5.66 (2.16;1.48)	0.05 (0.0040.51)	1.52 (0.80;2.90)	1.49 (0.63;3.53)	1.40 (0.56;3.40)	1.23 (0.77;1.98)	1.16 (0.50;2.66)	0.96 (0.41;2.23)
Neurologist	2.27 (1.10;4.70)	0.42 (0.13;1.33)	0.23 (0.17;0.72)	2.64 (1.14;6.11)	1.15 (0.09; 15.56)	0.32 (0.08;1.23)	3.01 (1.50;6.05)	2.40 (0.58;9.99)	2.75 (1.03;7.34)
Cardiologist	5.64 (4.17;7.62)	3.74 (1.65;8.45)	2.23 (0.88;5.60)	2.03 (1.38;2.97)	1.64 (0.90;2.96)	1.29 (0.76;2.16)	1.70 (1.24;2.32)	1.64 (0.91;2.98)	1.18 (0.75;1.87)
Psychiatrist	1.95 (0.96;3.96)	0.76 (0.36;1.56)	0.10 (0.03;0.42)	1.89 (0.99;3.60)	0.54 (0.12;2.34)	-	9.14 (4.78;17.44)	16.32 (2.09;127.37)	2.85 (0.52;15.59)
Physiotherapist	1.94 (1.00;3.76)	7.68 (2.03;28.9)	0.27 (0.09;0.77)	3.09 (1.51;6.31)	0.78 (0.17;3.49)	2.19 (0.68;7.03)	1.44 (0.77;2.71)	3.16 (0.81;12.31)	2.44 (0.86;6.89)
Psychologist	2.56 (1.0;6.49)	4.88 (1.04;22.9)	-	2.54 (1.02;6.32)	-	-	6.38 (4.15;9.80)	16.83 (2.18;129.86)	1.21 (0.30;4.91)

a) 95%CI: 95% Confidence interval; b) Emergency services: emergency care unit, private emergency room and emergency room; c) Specialist physicians: pulmonologist, neurologist, cardiologist and psychiatrist; d) Specialized services: physiotherapist and psychologist.

Notes: Pattern 1: hypertension, diabetes *mellitus* and cardiovascular diseases; Pattern 2: osteoporosis and rheumatism; Pattern 3: depression and anxiety.

Adjusted for sex, age (in years), marital status, income, hospitalization, body mass index (BMI) and smoking.

Refers to occasions when the sample size was small and it was not possible to perform the analyses.
